# School-family cognitive discrepancies in the cultivation of children’s interest in sports: an exploratory study based on grounded theory

**DOI:** 10.3389/fpubh.2025.1713106

**Published:** 2025-11-19

**Authors:** Haiou Zhong, Di Wang, Chunhua Qi

**Affiliations:** 1 Library, Chengdu Sport University, Chengdu, Sichuan, China; 2 School of Physical Education, Chengdu Sport University, Chengdu, Sichuan, China; 3 Chengdu Caotang Primary School, Chengdu, China

**Keywords:** children’s interest in sports, grounded theory, school-family cognitive discrepancies, sport education, overweight and obesity

## Abstract

**Objective:**

Physical activity is essential for children’s health, yet insufficient physical activity remains a global concern. Although interest in sports can enhance participation, cognitive discrepancies between School-Family in fostering that interest weaken its effectiveness, which is a significant issue. This study explores the manifestations and impacts of School-Family cognitive discrepancies in this process, aiming to identify intervention points for promoting children’s interest in sports, increasing physical activity, and preventing childhood overweight and obesity through School-Family collaboration.

**Methods:**

One-on-one interviews were conducted with 12 physical education teachers and 12 parents. A theoretical model of School-Family cognitive discrepancies in the cultivation of children’s sports interests was constructed based on grounded theory.

**Results:**

Five types of School-Family cognitive divergences are identified in the process of cultivating children’s interest in sports. Among these, Cognitive Biases in the Concept of Sports Education constitute the root cause, leading to Varying Strategies for Cultivating Children’s Interest in Sports, Inconsistent Perceptions of Physical Education Teaching Management, and Cognitive Differences in the Effectiveness of Sports Education. Cognitive Conflicts Regarding the Primary Responsibility for Sports Education functioned as an exacerbating factor that further intensified the disagreements regarding cultivation strategies, teaching management, and effectiveness evaluation.

**Conclusion:**

These studies preliminarily indicate that cognitive discrepancies can weaken the synergistic effects between schools and families in sports education practices, thereby reducing the effectiveness of fostering children’s interest in sports. This, in turn, leads to decreased physical activity among children and exacerbates childhood obesity and overweight. Interventions targeting key nodes within the model may provide a new evidence-based fulcrum for the prevention of overweight and obesity.

## 1 Introduction

The Annual Report on Physical Education and School Sport 2024 published by the Youth Sport Trust in the UK indicates that the physical condition of British children is a cause for concern, with one in five children being obese or overweight at the age of five (1). Similarly, the Guidelines for the Diagnosis and Treatment of obesity (2024 Edition) issued by the General Office of the National Health Commission of China point out that obesity has become a major public health issue. Among Chinese children and adolescents aged 6–17, the overweight rate and obesity prevalence are 11.1 and 7.9%, respectively; among those under 6 years old, the rates are 6.8 and 3.6%, respectively (2). To prevent and manage obesity and promote children’s physical health, the World Health Organization recommends that children engage in at least one hour of moderate-to-vigorous physical activity daily. However, these recommendations are often not met (3, 4). The root cause is children’s lack of children’s interest in sports during childhood, which results in low participation rates (5, 6). The implementation of public health prevention and intervention programs depends on a comprehensive understanding of all factors influencing the rates of overweight and obesity (7). Therefore, addressing how to cultivate children’s interest in sports, increase the physical activity participation rate, and reduce the rates of overweight and obesity is an urgent priority. Therefore, cultivating children’s interest in sports to enhance participation rates, reduce overweight and obesity prevalence, and promote holistic child development warrants significant attention. It should be particularly noted that, according to Article 1 of the Convention on the Rights of the Child, a child means every human being below the age of 18 years unless, under the law applicable to the child, majority is attained earlier (8). Based on this standard, the research subjects in this paper are clearly defined as children under the age of 18. Furthermore, since this study focuses on School-Family cognitive discrepancies—a key factor involving both the family and the school—in the text, children in the family environment are referred to as “children,” while those in the school context are referred to as “students”.

The cultivation of children’s interest in sports has also attracted the attention of scholars. However, existing studies have primarily focused on stimulating children’s individual interest and situational interest within classroom teaching (9, 10), while neglecting the importance of the family as a key agent outside the classroom (11). Both the family environment and the school environment are crucial factors in the formation and development of children’s interest in sports (12, 13). Parents, through their own physical activity demonstrations and verbal encouragement, can increase the probability of adolescents’ participation in sports by 85–156%, making them the primary factor influencing adolescents’ physical exercise behavior. In contrast, encouragement from physical education teachers can increase the probability of adolescents’ sports participation by 26%, positioning them as a secondary factor influencing adolescents’ physical exercise behavior (14). School-Family collaboration has been emphasized by many national education departments and basic education schools, becoming a research hotspot. Such collaboration not only helps improve the quality of physical education for children with disabilities (15), but can also serve as an effective tool to prevent childhood obesity, severe risk behaviors, and other health threats (16). Despite the significant positive implications of School-Family collaboration, the family and the school, as two distinct entities, often exhibit cognitive discrepancies (15, 17). In this study, “cognitive discrepancies” refer to the differing views held by families and schools regarding matters such as objectives, methods, and responsibility attribution in cultivating children’s interest in sports. These discrepancies may impede School-Family collaboration (17), thereby compromising the overall effectiveness of interest cultivation, reducing sports participation rates, and indirectly exacerbating childhood overweight and obesity. However, exploration into the specific dimensions of these cognitive discrepancies remains insufficient. Therefore, this research aims to systematically investigate the concrete composition and core manifestations of School-Family cognitive discrepancies in fostering children’s interest in sports. Given the complexity of this issue and the lack of relevant theories, grounded theory—which enables exploratory research by systematically analyzing raw data and constructing processual and narrative descriptions of specific phenomena—is employed in this study. This endeavor seeks to enhance the effectiveness of School-Family collaboration, thereby better nurturing and safeguarding children’s interest in sports, increasing sports participation rates, and preventing overweight and obesity. To the best of our knowledge, this is the first grounded theory study to explicitly elucidate “how these School-Family cognitive discrepancies, by weakening children’s interest in sports, serve as an upstream determinant of childhood obesity”.

## 2 Literature review

Cultivating children’s interest in sports arises from complex interactions between the child and multiple environmental systems. The ecological model for health promotion proposed by McLeroy et al. (18) posits that factors influencing health behaviors are distributed across multiple levels of influence: intrapersonal, interpersonal, organizational, community, and public policy. This model provides a macro-level framework for clarifying the influencing factors of children’s interest in sports. From this macro perspective, the family and the school, as the most immediate microsystems influencing the cultivation of children’s interest in sports, form a critical mesosystem through their interaction, thereby substantially impacting this developmental process (19).

### 2.1 Research on cultivating children’s interest in sports

Current research on cultivating children’s interest in sports primarily adopts two perspectives: fostering individual interest and developing situational interest. (1) Individual Interest. Existing studies have mainly focused on its influencing factors and its transformative relationship with situational interest. Regarding influencing factors, beyond actual skills, sports knowledge, perceived competence, and knowledge acquisition are identified as key internal mechanisms promoting the development of individual interest (9, 20). In terms of interest transformation, research confirms that situational interest can be effectively transformed into a stable individual interest through the design of the teaching environment (e.g., fostering a motivating atmosphere), with the two forming a dynamic developmental continuum (21, 22). Future research needs to further reveal the intrinsic mechanisms of this transformation process (23). (2) Situational Interest. Scholars generally recognize novelty, optimal challenge, attention demand, exploration intention, and instant enjoyment as the five core dimensions for cultivating situational interest (24). In practical teaching, educators can flexibly focus on one or two key dimensions for their instructional design, rather than attempting to integrate all (25). Specifically, while novelty and optimal challenge are dominant, their effects follow an inverted U-shaped curve, necessitating a gradual approach to avoid overstimulation (26–28). In contrast, instant enjoyment and exploration intention have consistently positive effects and emerge as key drivers for boys (29, 30). Furthermore, ensuring that students’ attention demands match their attentional capacity is a prerequisite for effective design (31). Beyond these five core dimensions, research has also explored innovative pedagogical approaches, such as using self-supervision videos and gamified teaching materials, to stimulate students’ situational interest in a multifaceted manner (32, 33).

### 2.2 Research on school-family cognitive discrepancies

The ecological model of health promotion emphasizes the critical importance of interaction and synergy among various systems, including the individual, interpersonal, organizational, community, and public policy levels (18). The Overlapping Spheres of Influence theory further posits that children’s development and educational outcomes are jointly shaped by three core domains: family, school, and community (34). While this theory outlines an ideal vision of school-family collaboration, such partnerships are often challenging to perfect in practice, largely because of the cognitive discrepancies deeply rooted between the perspectives of both parties. Existing research typically conceptualizes these discrepancies as clustering around three primary dimensions: (1) Divergence in Educational Goals and Values. Due to differing social roles and standpoints, families and schools hold fundamental differences regarding the ultimate aims of education. Schools, representing the national will and bearing the public mission of “fostering virtue and cultivating talents,” prioritize collective interests. In contrast, parents, representing individual educational philosophies, are primarily concerned with their child’s interests. While ensuring the child’s physical and mental health, they focus more intensely on securing a favorable position for the child in future society (35). This difference in value orientation is the deep-seated root of conflict. (2) Divergence in Teaching Strategies and Student Evaluation. At the practical level, significant discrepancies exist between the two parties regarding “how to teach” and “how to assess.” For instance, teachers tend to favor guiding students to explore independently to build self-confidence, whereas parents often prefer direct teacher intervention and precise tutoring to rapidly enhance academic performance (15, 17). (3) Divergence in Responsibility Boundaries and Communication Patterns. The aforementioned discrepancies further evolve into differing perceptions of educational responsibility attribution, leading to communication barriers (16, 36). Specifically, vague responsibility boundaries and the absence of an effective conflict-resolution mechanism create a situation where parents may expect frequent, direct communication, while schools might rely on an institutionalized channel. This fundamental discrepancy ultimately hinders deeper cooperation.

In summary, based on the research topic of “cultivating children’s interest in sports,” existing studies have primarily focused on the microsystem of the school, concentrating on in-class strategies for stimulating interest in sports. From the perspective of the research problem, while prior work has acknowledged the importance of school-family cognitive discrepancies as a key mesosystem factor, there is currently a lack of research specifically addressing these discrepancies in the context of cultivating children’s interest in sports. The formation of children’s interest in sports is profoundly influenced by their social and cultural environment (23), particularly the education provided by schools and the support from families (37). Family and school are recognized as the two most important environments closely related to child development (38). Although School-Family synergy is crucial, positive collaboration between them is not always easily achieved (39). Cognitive discrepancies often arise between parents and teachers due to differences in social status, role positioning, educational concepts, cultural literacy, and other multifaceted reasons (36, 40). Consequently, this study introduces the concept of School-Family cognitive discrepancies into this discussion in order to construct a theoretical model aimed at filling this research gap. By revealing the composition and impact of these discrepancies, this research seeks to provide a cognitive integration perspective for School-Family collaboration in fostering children’s interest in sports and to offer a theoretical basis for subsequent child health interventions through the construction of diversified collaborative models.

## 3 Research design, category refinement, and model construction

### 3.1 Research methodology

**Figure 1:**
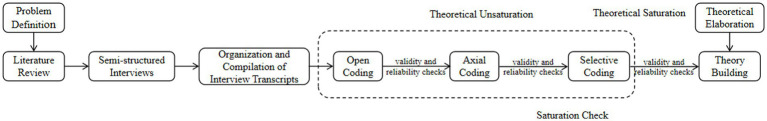
Research process.

Grounded theory, proposed by American scholars Barney Glaser and Anselm Strauss, is a methodology for theory discovery through systematic data collection and analysis (41). This study employs a grounded theory approach for two primary reasons. First, research on school-family cognitive discrepancies in cultivating children’s interest in sports is still in its early exploratory stage, lacking established theoretical frameworks. Correspondingly, a key strength of grounded theory is its ability to conduct exploratory research on novel or underexplored topics through the systematic organization and analysis from raw data (42). Second, school-family cognitive discrepancies involve two distinct entities (family and school), and the relational dynamics become particularly complex when the two parties hold conflicting views on educational decisions concerning the child (17). Grounded theory is uniquely suited as a methodological tool to effectively investigate such complex processes involving multi-stakeholder interactions and cognition. Therefore, grounded theory was selected for this study. The research procedure is illustrated in Figure 1.

### 3.2 Data collection

This study received ethical approval from the Ethics Committee of Chengdu Sport University (ChengTi LunLi 2025–145). All participants provided written informed consent, and the research team strictly adhered to protocols for protecting participant information. This study fully complies with the principles outlined in the 1964 Declaration of Helsinki and its subsequent amendments. Every effort was made to ensure the confidentiality and anonymity of participants, with strict measures in place to prevent any unauthorized disclosure of their private information.

This study adopted a theoretical sampling approach, that is, a purposeful sample selection method designed to propose a concept or construct a theory (43). Therefore, the selected samples are closely related to the research objectives. These samples are representative cases that can reflect certain phenomena, rather than statistically representative populations. Considering the diversity of entities involved in cultivating children’s interest in sports and the goal-orientation of the research, the following principles were followed in the selection of interview samples: (1) The theme of this study is the cultivation of children’s interest in sports, and the research question is about School-Family cognitive discrepancies. To ensure that the samples can cover the core fields and key entities in the cultivation of children’s interest in sports, school samples in this study were determined to be physical education teachers, and family samples were determined to be parents. (2) The ratio of teachers to parents among the interviewees should be balanced, and there should be certain differences in statistical characteristics such as educational background, gender, and age. (3) To ensure that the interviewees have certain experiential knowledge of the research questions, physical education teachers were required to have a teaching experience of at least 5 years. Parents were selected if their children were currently or had been in the school-age period. This is because children in this age group usually start to participate in school sports activities, and parents may have formed certain views and attitudes. (4) To avoid the interference of professional perspectives and focus on the general cognitive discrepancies in ordinary families, parents who are not engaged in sports-related occupations were selected. According to the “theoretical saturation principle” for determining the sample size in grounded theory (44), after each semi-structured interview was conducted, the interview data were immediately organized and analyzed. Samples were continuously selected until no new concepts could be extracted from the new samples (i.e., theoretical saturation was achieved). Finally, 24 interviewees were selected, including 12 physical education teachers and 12 parents. Table 1 shows the demographic characteristics of the samples, including gender, age, educational level, and role. The information of the interviewees is presented in Table 1.

**Table 1:** Basic demographic information of the interviewees.

Variable	Category	PE teacher (*N* = 12)		Parent (*N* = 12)	
		*N*	%	*N*	%
Gender	Male	4	33.33	7	58.33
	Female	8	66.67	5	41.67
Age	30 and under	6	50.00	2	16.67
	31–40	2	16.67	9	75.00
	Over 41	4	33.33	1	8.33
Education level	College or below	1	8.33	3	25.00
	Bachelor’s degree	4	33.33	8	66.67
	Graduate degree	7	58.33	1	8.33

The interviews were conducted in a semi-structured format, with questions dynamically adjusted during the process based on participants’ characteristics and their behaviors related to fostering children’s interest in sports. Each session lasted approximately 50 min. With participants’ consent, sessions were audio-recorded and transcribed verbatim. The interview guides were tailored for parents and physical education (PE) teachers (as shown in Table 2).

**Table 2:** Interview questions.

Parent version	Physical education teacher version
1 Do you think it is important for children to participate in sports activities? Why?	1 Do you think it is important for students to participate in sports activities? Why?
2 What sports activities have you arranged for your child? Why did you choose these specific sports activities instead of others?	2 What sports activities have been arranged for students at school? Why were these specific sports activities chosen instead of others? Do you think these arrangements are reasonable? Do you have any suggestions?
3 How do you cultivate your child’s interest in sports? Could you please elaborate on that?	3 How do you cultivate students’ interest in sports? Could you please elaborate on that?
4 In the cultivation of children’s interest in sports, who do you think should bear the primary responsibility—the family or the school? Why?	4 In the cultivation of students’ interest in sports, who do you think should bear the primary responsibility—the family or the school? Why?
5 Are you familiar with the school’s sports activity arrangements for your child? If so, what different ideas or opinions do you have regarding these arrangements?	5 During the cultivation of children’s interest in sports, do you have any different ideas or opinions from the students’ parents? How do you think the family and school can achieve better collaborative cooperation?

### 3.3 Category refinement and model construction

To enhance the accuracy and consistency of the coding process, this study adopted the researcher triangulation method (45). Two researchers initially conducted independent coding on a portion of the transcribed texts, followed by group discussions focused on the coding content. These discussions continued until a consensus was reached regarding the coding rules and conceptual definitions, thereby ensuring the reliability of the analytical results.

#### 3.3.1 Open coding

Open coding is the initial processing of raw data through word-, sentence-, and paragraph-level coding, labeling, and recording, aiming to identify valuable phenomena or events, extract initial concepts, and define conceptual categories (46). To ensure the authenticity of open coding, this study used the respondents’ original statements as the data source for mining initial concepts. After organizing the data collected from 24 interviewees, labels such as safety (parents), generality (PE teachers), focus on children’s physical conditions (PE teachers), and focus on children’s or parents’ preferences (parents) were derived. By retaining labels that appeared three or more times, 78 initial concepts and 13 categories were consolidated. Among them, the category “varied approaches to cultivating children’s interest in sports” was extracted through six concepts: parent–child sports activities (parents), teaching design (PE teachers), watching sports competitions (parents), participating in sports competitions (PE teachers), individual development (parents), and common development (PE teachers). Partial open coding results are presented in Table 3. This table illustrates the path from raw statements to concepts and categories by presenting verbatim quotes from different respondents in parallel.

**Table 3:** Excerpts from open coding.

Category	Conceptual code	Original statement
Different criteria for selecting sports activities	Preference of children or parents	“I choose sports for my child mainly by asking what they like and then signing up for that. Or, if I like something, I’ll have my child sign up for it too.” (Parent 1) “When I select an activity for my child, I’m the one who decides. I thought martial arts could teach self-defense, so I signed him up for it.” (Parent 3)
	Focus on children’s physical conditions	“When I select sports for students, I base it on their growth and development as well as their body proportions to determine what suits them best, and then I work on their psychological preparation.” (Teacher 8) “For some students who are overweight, I would suggest they start with activities like swimming, which places less stress on the joints.” (Teacher 3)
	Safety	“My main purpose in having my child learn sports is to strengthen their body and improve their health. I do not care what programs the school offers or what form the training takes, as long as the child does not get injured.” (Parent 4) “When selecting a sports program, my biggest concern is whether the venue and the coach are professional, and whether they can guarantee the child’s safety. Results are secondary.” (Parent 8)
	Generality	“What is taught in school is fixed; students practice general sports programs. It might be the same for an entire class, a grade, or even the whole school.” (Teacher 6)
Varied approaches to cultivating children’s interest in sports	Parent–child sports activities	“My main way of fostering my child’s interest in sports is to exercise together. For example, we do 30 sit-ups and 20 push-ups together after waking up in the morning and before going to bed at night.” (Parent 12) “On weekends, I take him to the park to ride a bike or simply play badminton downstairs. The key is that parents must participate and not just watch.” (Parent 6)
	Teaching design	“My main approach to fostering students’ interest in sports is to design some fun-based sports games during the teaching process.” (Teacher 8) “I adopt a tiered teaching approach, setting different goals and challenges for students with varying ability levels, so that everyone can experience a sense of success and thereby maintain their interest.” (Teacher 9)

#### 3.3.2 Axial coding

Axial coding aims to delineate the properties and dimensions of categories, discover their logical connections, and derive main categories (46). This study investigates School-Family cognitive discrepancies in fostering children’s interest in sports. Guided by their intrinsic logical connections, we synthesized five main categories from the conceptual-level data. The main categories and their corresponding initial categories are presented in Table 4. Specifically, Varied Criteria for Selecting Sports Activities, Diverse Approaches to, and Different Contents for, Cultivating Children’s Interest in Sports were consolidated under the main category “Varying Strategies for Cultivating Children’s Interest in Sports.” Similarly, differing views on physical education teaching philosophies, discrepant attitudes toward sports safety risks, and varied perspectives on sports resource allocation were grouped under the main category “inconsistent perceptions of physical education teaching management,” as all fall within the domain of instructional management.

**Table 4:** Axial coding.

Main category	Category
Z1 Varying strategies for cultivating children’s interest in sports	F1 Varied criteria for selecting sports activities
	F2 Varied approaches to cultivating children’s interest in sports
	F3 Different contents of children’s interest in sports cultivation
Z2 Inconsistent perceptions of physical education teaching management	F4 Differing views on physical education teaching philosophies
	F5 Discrepant attitudes toward sports safety risks
	F6 Varied Perspectives on Sports Resource Allocation
Z3 Cognitive conflicts regarding the primary responsibility for sports education	F7 Different views on the subject of sports safety responsibility
	F8 Cognitive discrepancies in the recognition of cultivation responsibility subjects
Z4 Cognitive differences in the effectiveness of sports education	F9 Inconsistent evaluations of sports training effects
	F10 Different views on the impact of sports on intelligence
Z5 Cognitive biases in the concept of sports education	F11 Differences in the recognition of sports importance
	F12 Disagreements in sports value concepts
	F13 Different purposes for cultivating children’s interest in sports

#### 3.3.3 Selective coding

Selective coding is the process of identifying a core category, via systematic analysis, from among all established categories. This core category then integrates all other categories into a coherent whole, encompassing most research findings within a broader theoretical framework (46). This study positions the cognitive divergences between school and family regarding the cultivation of children’s interest in sports as its core category.

**Figure 2:**
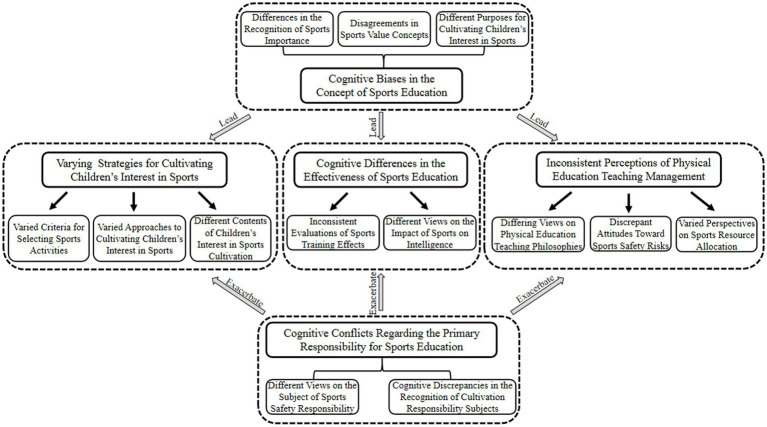
Theoretical model of school-family cognitive discrepancies in cultivating children’s interest in sports.

The storyline revolving around it unfolds as follows: The cultivation of children’s interest in sports relies on the close integration of physical and mental development, necessitating scientific guidance and School-Family collaboration to stimulate intrinsic motivation. However, cognitive biases in School-Family conceptions of sports education lead to Varying Strategies for Cultivating Children’s Interest in Sports, Inconsistent Perceptions of Physical Education Teaching Management, and Cognitive Differences in the Effectiveness of Sports Education. Cognitive conflicts between the two parties over primary responsibility exacerbate these challenges, which in turn undermines collaborative effectiveness and hinders the realization of synergistic outcomes. As shown in Figure 2.

#### 3.3.4 Theoretical saturation test

To ensure the scientific rigor of the grounded theory research process and the accuracy of the research findings, this study first conducted conceptualization of the implicit relationships among the concepts or categories formed through open coding and axial coding, as suggested by Glaser (47). Subsequently, by integrating relevant previous literature, the initially constructed theory and concepts were continuously compared with existing literature and concepts. Through repeated comparisons, no new conceptual dimensions emerged, indicating that theoretical and conceptual saturation had been achieved. Finally, using the same research procedures (i.e., coding and analysis), the theoretical model saturation was tested with the remaining one-third of the interview transcripts. The reserved eight interview transcripts were coded and analyzed following the same process as before. The results showed that the analysis of these interview data fully aligned with the previously identified relational attributes and conceptual dimensions. Specifically, no new main categories were identified through the coding and analysis of the last eight transcripts. When considering all the interview data collectively, they were all encompassed by the five main categories initially extracted. Accordingly, this study concludes that the selectively coded theoretical model has reached saturation.

## 4 Interpretation of the school-family cognitive divergence model in cultivating children’s interest in sports

### 4.1 Cognitive biases in the concept of sports education

Cognitive biases in the concept of sports education refer to the differing perspectives between schools and families regarding the importance of sports activities, core sports values, and the ultimate objectives of cultivating children’s interest in sports. These cognitive biases constitute the root cause of School-Family cognitive divergences in this domain, subsequently leading to Varying Strategies for Cultivating Children’s Interest in Sports, Inconsistent Perceptions of Physical Education Teaching Management, and Cognitive Differences in the Effectiveness of Sports Education.

Firstly, Differences in the Recognition of Sports Importance reflect divergent views on the role of sports in child development. In contemporary society, parents often prioritize academic achievement, inadvertently neglecting physical health and holistic growth. Some parents view sports as non-essential, equating the mere absence of illness with health and thus believing additional physical activity is unnecessary. As one parent candidly stated: “I do not think participating in sports is important. My child is very healthy. It’s better to spend that time memorizing more vocabulary words” (Parent 4). In stark contrast, physical education teachers emphasize the critical role of sports as a foundational component of holistic education.

Secondly, Disagreements in Sports Value Concepts stem from conflicting perceptions of the inherent value of sports. Many parents narrowly confine the purpose of sports to physical fitness—"enhancing physique and preventing diseases” (Parent 1). This perception often leads parents to discourage children from participating in activities perceived as risky. Physical education teachers, however, advocate for a broader educational perspective, asserting that sports are instrumental in comprehensively shaping character. As evidenced by prior research, sports cultivate children’s teamwork, perseverance, self-confidence, rule compliance, and social skills (48–50).

Finally, a significant disparity divides schools and families regarding their primary purposes for cultivating children’s interest in sports. Some parents pursue this goal with a short-term, flexible aim: hobby cultivation. “I primarily want my child to learn a sport they enjoy and gain one more hobby”(Parent 2). This approach reflects transient and adaptable characteristics—essentially, “participating when interested and withdrawing when not.” Conversely, schools prioritize achieving standardized physical fitness test outcomes, with a core focus on long-term sustainability and normative development.

Coordination between schools and families is paramount for educational success (51). As posited by Social Interdependence Theory, consistent goals foster positive interdependence, promoting trust and cooperation, whereas divergent goals create negative interdependence, leading to competition and obstruction (52–54). The cognitive biases in the concept of sports education establish a pattern of negative interdependence between schools and families. This not only undermines the efficacy of School-Family collaboration but also fragments children’s perceptions of sports values, ultimately diminishing children’s motivation to participate in sports activities.

### 4.2 Varying strategies for cultivating children’s interest in sports

To fully stimulate children’s interest in sports, schools and families often adopt different strategies based on their distinct perspectives. These varying strategies are both a consequence of cognitive biases in the concept of sports education and a direct manifestation of School-Family cognitive discrepancies in cultivating children’s interest in sports. Specifically, these differences are reflected in varied criteria for selecting sports activities, varied approaches to cultivation, and differences in the content of cultivation.

Firstly, parents’ decision-making tendencies when selecting sports activities exhibit two primary models. One model is based on children’s personal preferences, emphasizing a “child-centered” approach (55). This involves inquiring about children’s individual interest and respecting their autonomous choices to determine whether to pursue a particular sports activity. Although this child-centered educational philosophy is supported by many educators, it has also faced criticisms, such as the risk of becoming excessively centered on the child’s immediate desires (56). The other model reflects parents’ subjective intentions, where choices are made based on the parents’ own interests or experiences. Although these two models differ in focus, they often intertwine in practice, collectively influencing the cultivation of children’s interest in sports. In contrast, physical education teachers, from a professional standpoint, believe that sports selection should be based on students’ physical conditions. As one teacher noted: “When selecting sports activities, attention should be paid to students’ physical fitness and conditions. Different sports have different requirements, and suitable choices can maximize potential and avoid injuries” (Teacher 8).

Secondly, parents widely acknowledge the unique value of sports events in stimulating children’s interest. Therefore, taking children to watch various sports competitions has become a common strategy. They hope that the exciting scenes and emotionally charged atmosphere of competitions will provide strong sensory stimulation and emotional experiences, thereby igniting children’s curiosity and enduring passion for sports. “I often let my child watch sports games with me, whether online or offline, hoping that the atmosphere of the competition will cultivate their interest and love for sports” (Parent 7). Schools, however, focus on organizing students to actively participate in competitions, allowing them to experience the charm of sports through practice in competition and cooperation, thereby fostering a lifelong interest in sports. The renowned American educator John Dewey believed that education is not merely the transmission of knowledge but should cultivate students’ interests and abilities through practice (57). Participation in competitions is an excellent form of such practice, effectively generating situational interest.

Finally, regarding cultivation content, parents tend to expose children to a wide range of sports activities to explore their interests. As one parent stated: “Water sports, land sports, team sports, individual sports—let the child experience all kinds of activities to see which one they like” (Parent 9). However, since physical education test scores are a key quantitative metric for evaluating the effectiveness of school sports education, schools often devote more effort to training for test items such as standing long jump, running, and rope skipping. The practice of test items tends to be uniform, repetitive, and monotonous, leading students to develop aversion toward sports and gradually lose interest in participating in physical activities (58).

According to Self-Determination Theory (59), children’s intrinsic motivation (e.g., interest in sports) and external environment (e.g., family and school support) jointly influence their behavioral performance. Intrinsic motivation, stemming from the satisfaction of autonomy and competence derived from the activity itself, is the core driver of sustained participation in sports. The external environment can stimulate or maintain motivation and promote positive behavior by providing supportive conditions that satisfy these basic psychological needs. However, when families emphasize “autonomous interest” while schools focus on “external evaluation,” children receive conflicting messages: the family supports autonomy, but the school environment may suppress it. This strategic conflict prevents children from experiencing autonomous enjoyment while also making it difficult for them to receive competence feedback, ultimately leading to decreased enthusiasm for sports participation or even avoidance of physical activities. Additionally, as most parents have relatively limited knowledge of sports compared to physical education teachers, children may end up participating in sports unsuitable for their physical conditions, thereby increasing the risk of sports injuries and ultimately undermining their interest in sports.

### 4.3 Inconsistent perceptions of physical education teaching management

Beyond the varying strategies for cultivating children’s interest in sports, cognitive divergences in the concept of sports education also lead to inconsistent perceptions of physical education teaching management. Specifically, schools and families hold different views regarding teaching philosophies, safety management, and resource allocation within physical education. These inconsistent perceptions represent a practical barrier to School-Family collaboration in fostering children’s interest in sports.

Firstly, differing views on physical education teaching philosophies primarily reflect disagreements between families and schools over “how to teach” revealing an inherent tension in their approaches to instructional practice. Physical education teachers often tend to be strict and critical toward students to achieve better teaching outcomes, whereas parents believe the teaching process should emphasize positive reinforcement and encouragement. Existing research indicates that to encourage children’s active participation in sports, coaches should employ more positive rather than negative behaviors (60). Greater encouragement and support enable children to better enjoy the sports process and cultivate a sustained interest in sports.

Secondly, the physical education curriculum is a key component of teaching resources, and there is disagreement between families and schools regarding the “quantity” of such classes. “There are too many physical education classes—five sessions a week, even exceeding the number of English classes” (Parent 8).

Parents expressed strong dissatisfaction, which may stem from their prioritization of academic subjects and underestimation of the value of physical education. In contrast, physical education teachers perceive the current allocation of classes as insufficient in both time and resources. “The number of physical education classes is not excessive. Each session lasts only 40 min, and due to large class sizes, it is difficult to provide adequate guidance to every student” (Teacher 7).

Finally, discrepant attitudes toward sports safety risks refer to the differing perspectives of parents and schools regarding safety management in children’s physical activities. Specifically, parents may tend to restrict their children’s exercise intensity or opt for low-risk sports due to concerns about potential injuries. Schools, however, often emphasize the educational value and holistic development offered by physical activities, viewing moderate risk as an inevitable aspect of physical education, thus leading to divergent approaches and attitudes toward safety management.

Existing research suggests that cognitive discrepancies between teachers and parents can easily escalate into conflicts, undermining collaboration and directly inhibiting children’s development (17). In the process of cultivating children’s interest in sports, School-Family disagreements over the management of physical education—particularly regarding attitudes toward safety risks—pose significant challenges. For instance, parents may intervene in school physical activities due to concerns about sports-related injuries. While such protective interventions may mitigate short-term risks, they can cause children to miss critical periods for motor skill development, lead to the cancellation of valuable high-intensity physical activities, and ultimately restrict opportunities for children to explore diverse sports interests, thereby hindering the comprehensive cultivation of children’s interest in sports.

### 4.4 Cognitive conflicts regarding the primary responsibility for sports

Education Cognitive conflicts regarding the primary responsibility for sports education (including different views on the subject of sports safety responsibility and cognitive discrepancies in the recognition of cultivation responsibility subjects) refer to the conflicting perceptions between families and schools concerning the attribution of responsibility for sports education. These conflicts act as an exacerbating factor in School-Family cognitive discrepancies regarding the cultivation of children’s interest in sports. They intensify the existing divergences in strategies for cultivating children’s interest in sports, inconsistent perceptions of physical education teaching management, and cognitive differences in the effectiveness of sports education.

Different views on the subject of sports safety responsibility indicate inconsistent opinions between families and schools regarding who should bear the safety responsibility for children during sports activities, leading to ambiguity in responsibility allocation. When safety incidents occur in physical education classes, parents often blame the physical education teachers. In contrast, physical education teachers argue that: “they have fulfilled their duty of reminder, but some students still fail to comply with safety rules, leading to accidents, and thus it is not entirely their responsibility” (Teacher 10). Although relevant legal provisions stipulate that schools and teachers should not bear full responsibility for injuries caused by students’ own actions after fulfilling reasonable reminder and safety assurance obligations (61), some parents still resort to irrational means to hold schools accountable (Teacher 3). Due to the fear of sports safety incidents, physical education classes have evolved into a “three-noes and seven-nots” pattern: no intensity, no difficulty, no confrontation, no sweating, no panting, no running, no dirty clothes, no falling, no skin abrasions, and no sprains (62).

Furthermore, while children’s education should be a shared responsibility between parents and teachers (63), in practice, responsibility-shifting often occurs (64). This phenomenon is particularly evident in the cultivation of children’s interest in sports. For instance, one parent stated: “Regarding the cultivation of children’s interest in sports, I believe the school is the primary responsible party because children spend most of their time there” (Parent 7). However, physical education teachers emphasize that although schools serve as the main arena for cultivating children’s interest in sports, effectively addressing this issue requires joint efforts with families. Research indicates that the social mechanisms influencing children’s sports interests and behaviors are complex and extensive, with parents being one of the key factors (65). Parental sports interests, philosophies, and physical exercise behaviors significantly impact children’s exercise frequency, duration, and intensity (66).

The Overlapping Spheres of Influence theory (34) emphasizes that families and schools, as two critical environments for children’s development, consistently interact and permeate each other’s roles, sharing consistent goals and jointly bearing the responsibility for children’s education. This implies that the responsibility for children’s sports education cannot be solely attributed to either schools or families but requires collaborative efforts and shared accountability from both parties. However, in practical cooperation, families and schools often tend to evade their own responsibilities or shift them onto the other party. Cognitive conflicts regarding the primary responsibility for sports education not only intensify the contradictions in School-Family collaboration for cultivating children’s interest in sports and impair the atmosphere and effectiveness of such cooperation but may also lead to overly conservative physical education curricula due to safety concerns. This results in programs lacking challenge and fun, ultimately undermining children’s interest in sports.

### 4.5 Cognitive differences in the effectiveness of sports education

Cognitive Differences in the Effectiveness of Sports Education refer to significant School-Family disagreements in evaluating sports training effectiveness and its impact on students’ intellectual development. These differences represent both the outcome and feedback of School-Family discrepancies in cultivating children’s interest in sports and biases in physical education concepts.

This study reveals substantial cognitive biases in School-Family understanding of sports training effectiveness. Specifically, some parents adhere to an intuitive “sweat index theory,” considering sweat volume as a direct effectiveness indicator. For example, one teacher reported: “After a physical education class, a parent asked me, ‘Teacher, the students did not even sweat during today’s training—was it effective?’” (Teacher 7). This parental perspective simplifies physical activity understanding by equating sweat with exertion. However, physical education teachers argue that effectiveness should not be judged solely by sweat volume. The notion that “more sweat indicates better results” lacks scientific basis, as sweating is not an evaluation criterion (67). Holistic evaluation of training effectiveness should consider exercise purposes, methods, duration, intensity, and physiological adaptation.

More critically, significant disagreements exist regarding whether sports promote intellectual development, particularly whether physical activities hinder academic performance. “Physical activities can negatively impact academic performance” (Parent 6). Parental concerns are primarily reflected in two aspects: firstly, physical activities may encroach on the time allocated to academic subjects; secondly, the fatigue induced by sports participation may impair the effectiveness of academic learning. Conversely, physical education teachers maintain that sports positively influence academic performance. Regarding whether physical activity affects academic performance, research indicates that physical activity can enhance academic achievement (68). Instead, by enhancing executive functions—a key pathway—it can establish a solid cognitive foundation for academic success, thereby generating positive effects (69, 70). However, the extent of this impact is moderated by factors such as the duration and content of the physical activity, as well as the methods used to assess academic performance. This suggests that the effect of physical activity on academic achievement may vary depending on specific contexts and intervention designs (71).

Although children benefit profoundly from sports engagement (48–50, 72), dropout rates remain high (73, 74). As this study finds, some parents still question effectiveness, primarily concerned about academic decline, consequently withholding support. These cognitive differences cause parental reservations or misguidance in encouraging sports participation, hindering positive attitude development and sustained interest, while impeding school physical education advancement and children’s holistic development. Consequently, schools and parents should collaborate through scientific education and communication to eliminate these differences, ensuring children gain comprehensive development through sports activities. This collaborative approach ultimately builds a favorable School-Family atmosphere conducive to cultivating children’s interest in sports.

## 5 Conclusions and prospects

### 5.1 Conclusion

Employing a grounded theory approach, this study conducted systematic analysis and progressive coding of textual data collected through semi-structured interviews, yielding the following conclusions: (1) School-Family cognitive divergences regarding the cultivation of children’s interest in sports encompass Cognitive Biases in the Concept of Sports Education, Varying Strategies for Cultivating Children’s Interest in Sports, Inconsistent Perceptions of Physical Education Teaching Management, Cognitive Conflicts Regarding the Primary Responsibility for Sports Education, and Cognitive Differences in the Effectiveness of Sports Education. (2) Cognitive Biases in the Concept of Sports Education between schools and families lead to Varying Strategies for Cultivating Children’s Interest in Sports, Inconsistent Perceptions of Physical Education Teaching Management, and Cognitive Differences in the Effectiveness of Sports Education. These divergences are further exacerbated by Cognitive Conflicts Regarding the Primary Responsibility for Sports Education between the two parties, thereby undermining the effectiveness of their collaboration in sports education practice and hindering the full realization of synergistic outcomes. This creates an impact pathway of “cognitive divergences → weakened collaboration → diminished interest → reduced participation → increased obesity.” (3) School-Family cognitive divergences concerning the cultivation of children’s interest in sports represent upstream determinants of childhood obesity and overweight, as well as relevant modifiable environmental determinants. Within the model of these cognitive divergences, “Cognitive Biases in the Concept of Sports Education” and “Cognitive Conflicts Regarding the Primary Responsibility for Sports Education” constitute critical intervention points.

### 5.2 Research contributions

#### 5.2.1 Innovations in research content

Current academic research on cultivating children’s interest in sports primarily focuses on stimulating individual and situational interest within classroom teaching contexts. This study breaks through this limitation by extending the research perspective to the “front-end of School-Family interaction” for the first time. Employing grounded theory methodology, it systematically identifies and categorizes five types of cognitive divergences between families and schools in this process, specifically including: (1) Cognitive Biases in the Concept of Sports Education; (2) Varying Strategies for Cultivating Children’s Interest in Sports; (3) Inconsistent Perceptions of Physical Education Teaching Management; (4) Cognitive Conflicts Regarding the Primary Responsibility for Sports Education; and (5) Cognitive Differences in the Effectiveness of Sports Education. Crucially, these cognitive divergences are explicitly defined as “modifiable environmental determinants” influencing childhood overweight and obesity, thereby providing clear and actionable targets for practical interventions.

#### 5.2.2 Theoretical innovations

This study constructs a theoretical model of School-Family cognitive divergences in cultivating children’s interest in sports and for the first time reveals the impact pathway: “cognitive divergences → weakened collaboration → diminished interest → reduced participation → increased obesity risk.” It incorporates “School-Family cognitive divergences” as an integral contextual-level construct into child health promotion theory and identifies two core drivers: “Cognitive Biases in the Concept of Sports Education” and “Cognitive Conflicts Regarding the Primary Responsibility for Sports Education.” This model addresses a critical gap in research on family-school interaction mechanisms in childhood obesity prevention, establishing a novel theoretical framework supported by empirical evidence for upstream interventions.

### 5.3 Research implications

The School-Family cognitive discrepancy model constructed in this study provides a theoretical basis and operational pathway for designing targeted public health education interventions. Future work can focus on the following three core directions: First, implement layered interventions targeting the core sources of conceptual and responsibility-based discrepancies. To address cognitive biases in the concept of sports education, designing “School-Family Sports Education Consensus Workshops” that utilize structured debates and scenario simulations could facilitate mutual understanding and two-way calibration on issues such as the importance of sports activities, sports values, and the purposes of cultivating children’s interest in sports. Regarding cognitive conflicts over the primary responsibility for sports education, at the policy level, educational administrative departments should take the lead in formulating Guidelines for School-Family Shared Responsibility in Sports Safety, clarifying the boundaries of “duty of care” and standardizing accident handling procedures. Building on this, schools and families should be encouraged to jointly develop a “Shared Responsibility Agreement for Children’s Sports Health,” delineating responsibilities and collaborative processes for families and schools concerning safety, resources, and interest cultivation, thereby transforming disputes over responsibility into a cooperative framework. Second, develop practical programs to bridge strategic choice discrepancies. For instance, to reconcile the strategic preferences of families for “interest exploration” versus schools for “skill mastery,” implementing “Sports Homework” (75) could be effective. Its design should integrate enjoyable parent–child interactions with structured skill practice to achieve a win-win outcome. Additionally, creating a “Sports Activity Selection Advisory Platform,” guided by professional teachers, could help parents make scientific choices based on children’s physical condition and interest profiles, reducing the risk of activity mismatch from the outset. Third, establish an assessment system that supports school-family collaboration. This involves shifting the evaluation paradigm from singular judgment to communicative intervention and building an evidence-based effectiveness communication mechanism. For example, by developing learning outcome feedback tools and sharing data on the correlation between sports participation and academic performance, assessment results can be transformed into a cornerstone for communication. This would visually demonstrate training effects and directly address parental concerns, thereby addressing cognitive differences in the effectiveness of sports education.

### 5.4 Limitations and future research

#### 5.4.1 Limitations of sample and research prospects

This study acknowledges that, as the sample was exclusively drawn from Chengdu, China, the generalizability of its findings to other regions in China or to different cultural contexts may be limited. However, this limitation renders Chengdu an ideal case for an in-depth analysis of how specific socio-cultural factors in China shape School-Family cognitive discrepancies in cultivating children’s interest in sports. As a core city and major educational hub in Western China, Chengdu’s educational ecosystem encapsulates the typical characteristics of School-Family interactions in China. Our grounded theory analysis identified three fundamental drivers within the Chinese context: the highly competitive educational culture, unique family structures, and the secondary school physical education entrance exam policy. These drivers systematically generate and perpetuate the five cognitive discrepancies. For instance, the culture of “academic primacy” directly exacerbates Cognitive Biases in the Concept of Sports Education, while the “intensive parenting” model, stemming from the long-standing one-child policy, profoundly influences Discrepant Attitudes Toward Sports Safety Risks between schools and families. The convergence of these factors creates a “high-intensity” context that amplifies and brings into sharp relief the tensions inherent in School-Family interactions. Therefore, the value of this model lies in the general mechanisms it reveals, rather than in its region-specific manifestations. The five categories of cognitive discrepancies identified by the model—conceptual, strategic, instructional, responsibility, and effectiveness—constitute a fundamental analytical framework for understanding School-Family interactions in sports, offering theoretical insights that transcend specific contexts. We hypothesize that while the basic structure of the model remains robust across different settings, the specific manifestations and interaction intensities of each dimension may vary with local conditions. Future research should conduct cross-contextual comparisons on a broader scale to validate and refine this theoretical framework, thereby clarifying its boundary conditions.

#### 5.4.2 Additional limitations and future research

While this study ensured comprehensiveness in data collection and coding, adhering to theoretical saturation, interview texts inherently contain certain biases. Future research could employ the Delphi method to further validate and supplement the theoretical model of School-Family cognitive discrepancies in cultivating children’s interest in sports. A second limitation concerns gender effects. While this study primarily focused on School-Family cognitive discrepancies, it did not deeply analyze the potential moderating effect of children’s gender on the manifestation and intensity of these discrepancies. Societal gender norms may predispose both families and schools to form differentiated sports expectations and risk perceptions for children of different genders, thereby shaping the cognitive discrepancies in distinct patterns. Future studies should integrate children’s gender into the analytical framework to explore the complexity of how these discrepancies operate, providing a foundation for developing more targeted, group-specific interventions. Furthermore, although this study theoretically explored School-Family cognitive discrepancies based on the authentic experiences of physical education teachers and parents, related theoretical research in this area remains scarce. Given the significant impact of these cognitive discrepancies on cultivating children’s interest in sports, more theoretical guidance is essential to better promote School-Family collaboration. Finally, the pathways of influence proposed in this study have not yet been quantitatively validated. Subsequent research should develop scales and conduct large-scale empirical studies to test these pathways and provide data-driven support for intervention strategies.

## Data Availability

The raw data supporting the conclusions of this article will be made available by the authors, without undue reservation.
